# Supplementary material for: Implicit neural representations for accurate estimation of the Standard Model of white matter
Source: Commun Biol. 2025 Dec 24;9:120. doi: 10.1038/s42003-025-09399-5 (PMC12852660; doi:10.1038/s42003-025-09399-5)
Supplement: Supplementary file 1 — Supplementary material [file 42003_2025_9399_MOESM1_ESM.pdf]

Supplementary Material

Implicit neural representations for accurate estimation of the Standard Model of white matter

1 Choice of hyperparameter σ^2

The Fourier Feature (1) encoding provides an additional hyperparameter σ^2 that controls the width of the normal distribution where the encoding frequencies are sampled from. This provides extra flexibility to the user to control the spatial regularization of the representation. Lower σ^2 yields lower frequencies, which capture less detail and noise, while higher σ^2 does the opposite. This implies that downstream tasks which do not require exceptionally fine details could benefit from lower σ^2 to mitigate noise, while higher quality datasets can be modeled with higher σ^2 to maintain contrast in regions with high frequency changes in signal.

To illustrate the effect σ^2 has on the representation, we fit an INR on the synthetic datasets with Gaussian noise SNR 50 and 20 for each $\sigma^2 \in \{1, 2, 4, 8\}$. The D_i parameter map is visualized for every INR in Figure S1.

At $\sigma^2 = 1$ we see an overly smooth representation, losing some finer detail. These details start to re-appear at $\sigma^2 = 2$. While for SNR 50 the $\sigma^2 = 4$ still provides a good representation, for SNR 20 the INR starts capturing too much of the noise. For $\sigma^2 = 8$ both datasets produce an overly granular parameter map. In general we would recommend starting with $\sigma^2 = 3.5$, and move up or down based on downstream requirements and dataset quality.

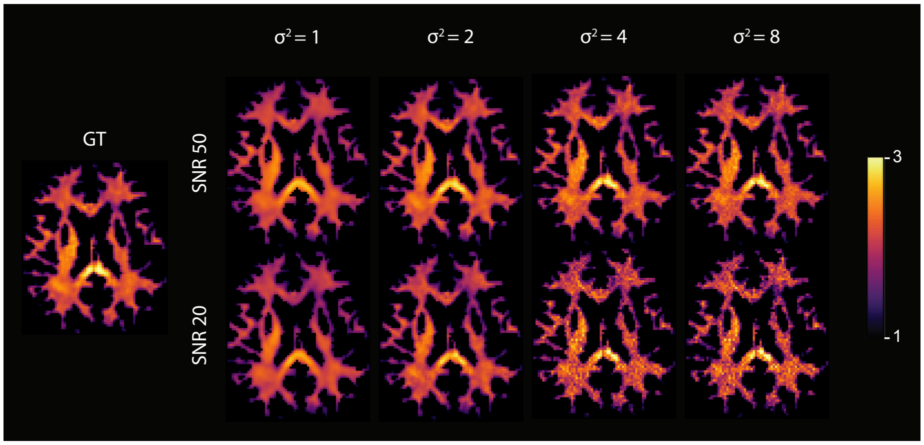

Figure S1: Parameter maps of D_i for the INR fit on the synthetic datasets with SNR 50 and 20 (rows) with varying σ^2 (columns)

2 Noise comparison

Scatter density plots from Fig.S2a demonstrate that the most accurate voxel-wise estimates are obtained with NLLS in the noiseless case as expected, where $\rho > 0.999$. Only RMSE of p_2 deviates notably, which is attributed to the lack of constraints on the fODF coefficient values in the NLLS method, leading to outliers. The supervised NN also shows high correlation for the kernel ($\rho > 0.995$) but shows low accuracy for p_2. The INR shows the lowest accuracy for f_i and $D_e^{\perp}$. SMI performs very accurately for the bulk of the voxels, e.g. less broad scatter density plots than the INR, but shows high RMSE values for D_i and $D_e^{\parallel}$ due to outliers at low ground truth diffusivity values. Visual inspection of the brain parameter maps in Fig. S2b shows that all fitting methods are capable of producing smooth parameter maps, similar to the ground truth.

Fig.S3 shows results for experiment 1 where Gaussian noise is considered with SNR 50. The INR method shows to be noise robust also at higher noise levels and outperforms the other methods. Note the spatial smoothness of the parameter maps estimated by the INR method compared to the other very noisy maps.

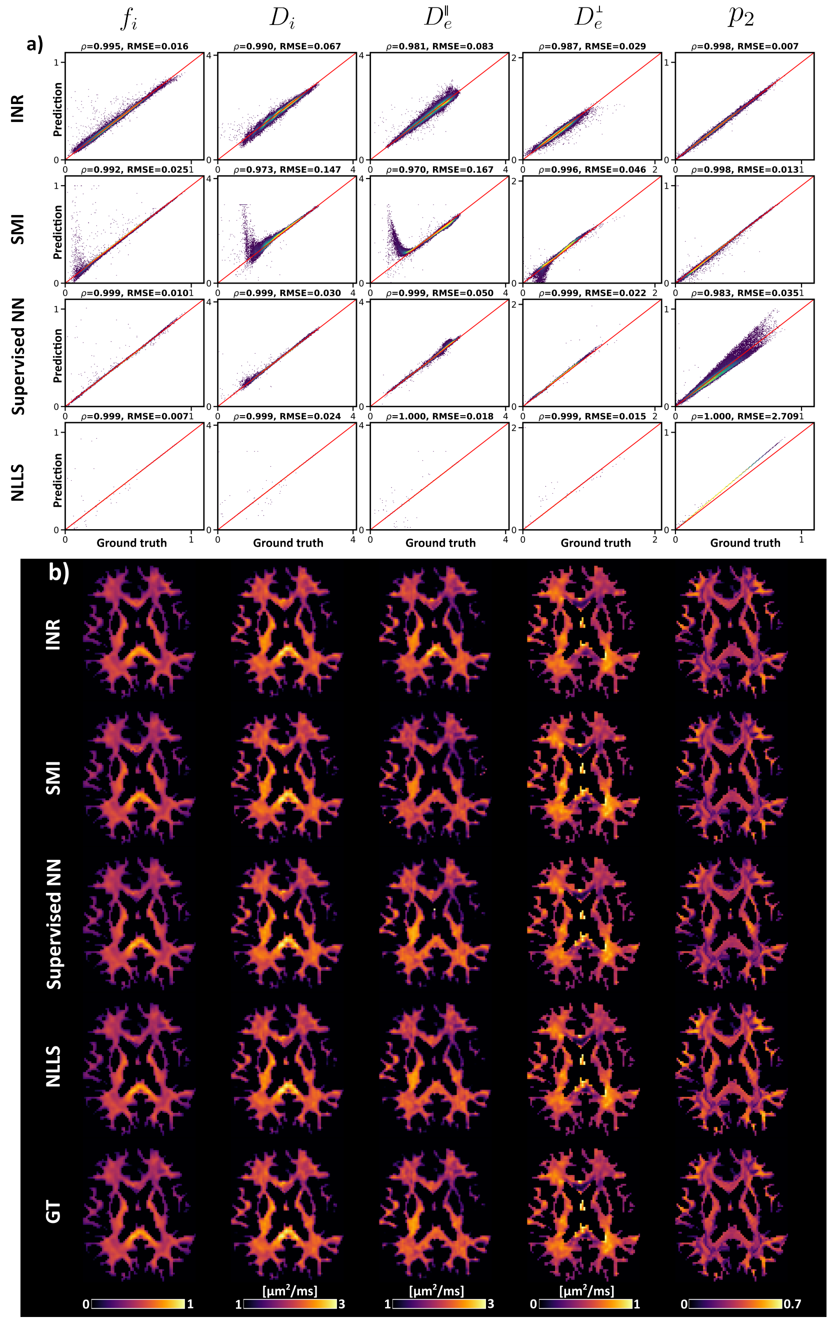

Figure S2: Experiment 1 (No Noise): **a)** Scatter density plots of the ground truth versus the parameter estimations of all methods. Row numbers correspond to method numbering from section 2.5 of main text. The titles of the subplots indicate ρ and RMSE. **b)** SM parameter maps corresponding to the results in **a**. Bottom row shows the ground truth (GT).

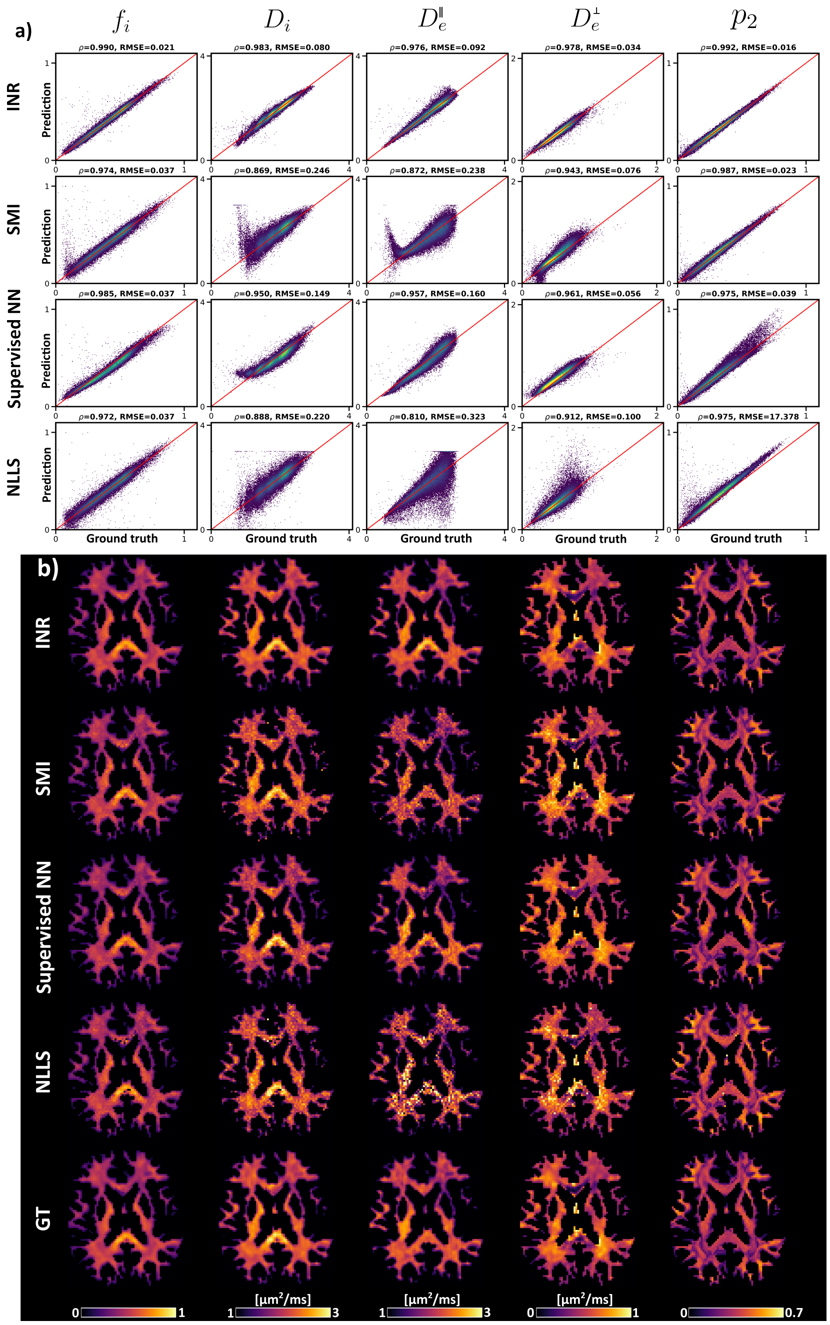

Figure S3: Experiment 1 (SNR 50): **a)** Scatter density plots of the ground truth versus the parameter estimations of all methods. Row numbers correspond to method numbering from section 2.5 of the main article. Title of the subplots indicate ρ and RMSE. **b)** SM parameter maps corresponding to the results in **a**. Bottom row shows the ground truth (GT).

3 Rician loss SNR 50

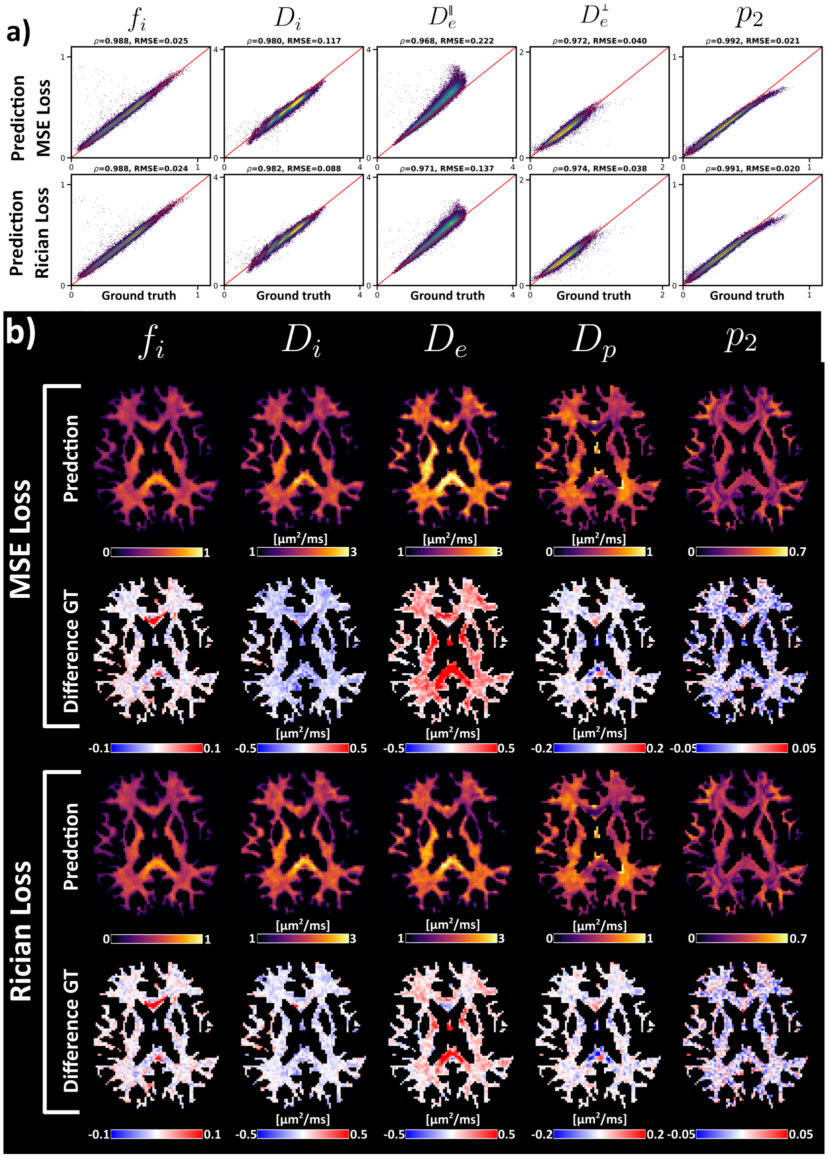

Figure S4: Effect of Rician Loss Likelihood function (SNR = 50). **a)** Scatter plots for estimation with MSE and Rician loss. **b)** Brain parameter maps of the predictions from **a**. Difference maps are with the ground truth parameters.

4 Parameter estimates for higher SH orders

To illustrate the difference in parameter estimates between different l_{max} values, we calculate the median absolute difference between parameter maps. For the synthetic datasets this is calculated between the ground truth parameter maps and the different SH order fits of the INR, the results of which are shown in Table S1. Because there is no ground truth available for the real dataset, we compare the higher SH order estimates with the $l_{max} = 2$ fit. These results are shown in Table S2. For both the synthetic and the real dataset there are no notable differences between the different SH order estimates. The noise robustness of the model is shown in the synthetic dataset, where the difference with the ground truth increases only slightly in the SNR = 50 dataset, compared to the noiseless one.

	l_{max}	f_i	D_i	$D_e^{\parallel}$	$D_e^{\perp}$	S_0	p_2
Noiseless	2	0.006	0.034	0.046	0.015	0.728	0.004
	4	0.006	0.034	0.045	0.015	0.794	0.005
	6	0.006	0.034	0.044	0.014	0.629	0.005
	8	0.006	0.034	0.043	0.014	0.547	0.005
SNR = 50	2	0.011	0.047	0.051	0.019	1.628	0.010
	4	0.010	0.046	0.052	0.019	1.696	0.011
	6	0.010	0.046	0.051	0.019	1.855	0.011
	8	0.010	0.046	0.051	0.019	1.643	0.011
SNR = 20	2	0.019	0.083	0.130	0.032	3.674	0.023
	4	0.017	0.071	0.166	0.037	3.494	0.026
	6	0.018	0.068	0.148	0.035	3.451	0.028
	8	0.020	0.077	0.129	0.034	3.403	0.029

Table S1: Table showing the median absolute differences between the ground truth and two synthetic dataset (noiseless and SNR = 50 gaussian noise) INR fits for different SH orders (l_{max}).

	l_{max}	f_i	D_i	$D_e^{\parallel}$	$D_e^{\perp}$	S_0	p_2
Real	2	0.000	0.000	0.000	0.000	0.000	0.000
	4	0.005	0.016	0.022	0.011	5.725	0.003
	6	0.006	0.019	0.033	0.015	5.707	0.003
	8	0.006	0.018	0.033	0.015	4.419	0.003

Table S2: Table showing the median absolute differences between the $l_{max} = 2$ INR fit for the real dataset and the higher SH order INR fits.

5 Rician loss with gradient non-uniformtiy correction

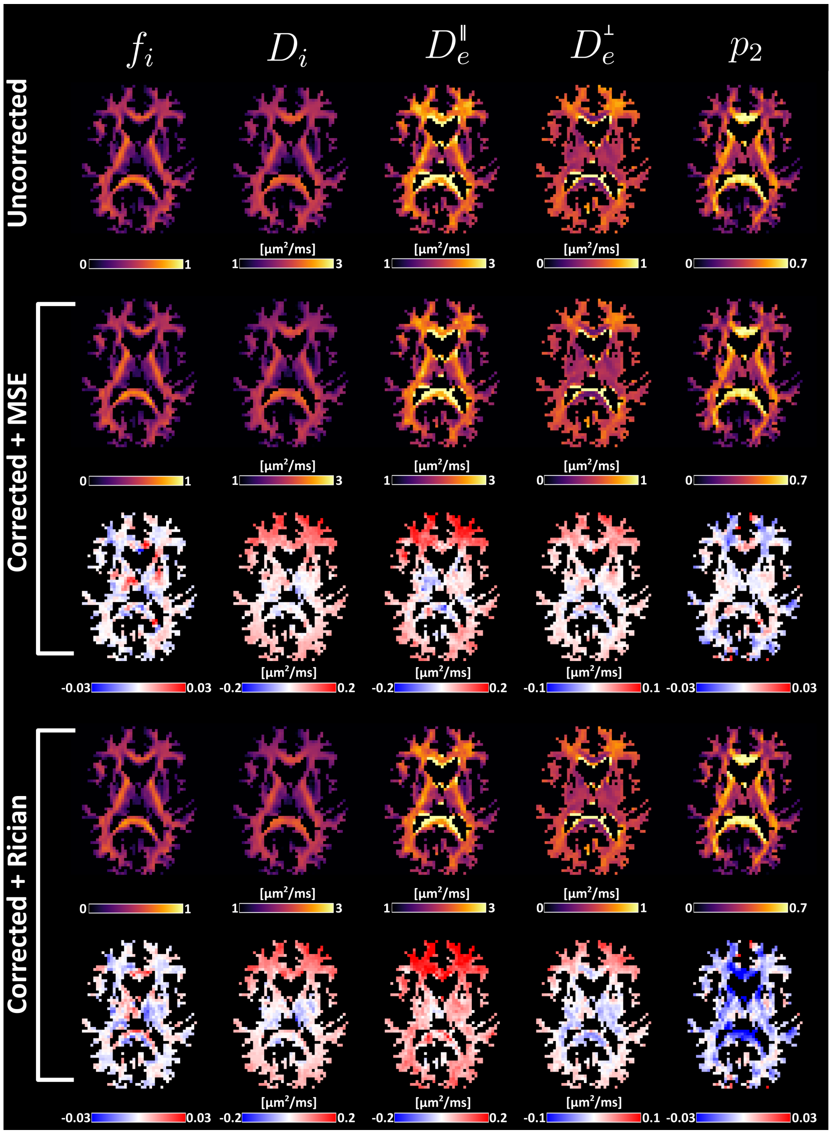

Figure S5: Effect of gradient non-uniformity correction on the estimation of SM parameters. Top row shows parameter maps without correction. Bottom rows show the effect of gradient non-uniformity correction with MSE loss and Rician loss. Difference maps are with respect to prediction with MSE and without gradient non-uniformity correction (top row).

6 Generation of ground truth

The procedure for generating the ground truth is outlined in detail in Section 2.5 of the main text. It should be noted, however, that the generation of such a dataset is not governed by a rigid stepwise protocol. Instead, several methodological choices are required, each of which may influence the resulting ground truth and, consequently, the outcomes of analyses that rely on it for validation of different fitting methods. To elucidate the implications of these decisions, this section examines the effect of specific choices on the ground truth generation process.

First, the choice of dataset forming the basis of the ground truth is of particular importance. In this work, subject 11 from the MGH Connectome Diffusion Microstructure (CDMD) dataset was selected. The DWIs acquired with $\Delta = 19$ ms were used and fitted with SMI to generate the ground truth, as relaxation effects were not considered and thus a single acquisition time had to be chosen. The DWI's with $\Delta = 49$ ms could potentially be more appropriate for the SM due to the longer diffusion time and hence it's influence is investigated.

Second, the use of a noise variance (sigma) map during SMI fitting may introduce bias in the ground truth generation. The standard approach in this study employed a sigma map estimated with MPPCA. However, this method may underestimate the noise level, as the MGH CDMD dataset is preprocessed but not denoised, which introduces correlations in the noise and violates the underlying assumptions of MPPCA. To assess the impact of this, alternative noise estimates were explored, including using a standard deviation–based sigma map from the $b0$ acquisitions ($\Delta = 49$ ms, method 1), using an MPPCA-based sigma map derived only from the $b0$ acquisitions ($\Delta = 49$ ms, method 2), and omitting a sigma map ($\Delta = 49$ ms, method 3).

Third, the effect of excluding the $b = 200$ and $b > 10{,}000$ acquisitions was evaluated. The $b = 200$ acquisitions may introduce free water contributions to the signal, while the $b > 10{,}000$ acquisitions may suffer from insufficient SNR ($\Delta = 49$ms method 4) .

The results are presented as histograms and scatter plots of the parameter distributions obtained from the different SMI fits. Figure S6 shows scatter plots comparing the alternative settings with the reference settings. Figure S7 presents histograms of the resulting parameter distributions. Finally, Figure S8 shows the fitting performance of the methods on simulated data without smoothing, using a noise level of SNR = 50.

The results indicate that the parameter distributions are only minimally affected by the different settings when using the $\Delta = 49$ ms acquisition. The observed variation in the scatter plots can largely be attributed to degeneracies of the SM when fitted with LTE acquisition schemes. Figure S8 indicates no

bias introduced by the smoothing kernel since the method comparison shows similar trends to those obtained with smoothed ground truth; specifically, the INR method continues to outperform the other approaches. The performance of INR on non-smoothed simulated data is lower compared to the results with smoothed data, which is expected given that the method relies on spatial correlations to enhance fitting performance.

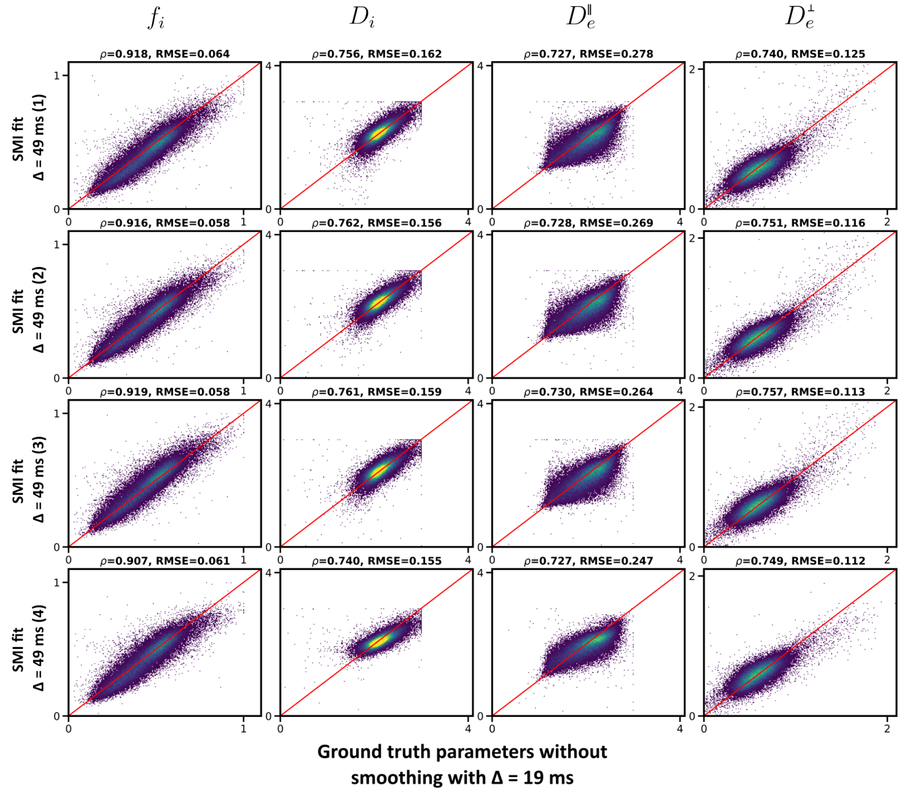

Figure S6: Scatter plots of SM parameter distributions, with the y-axis showing results obtained using alternative settings and the x-axis showing results obtained with the reference settings used in the main document.

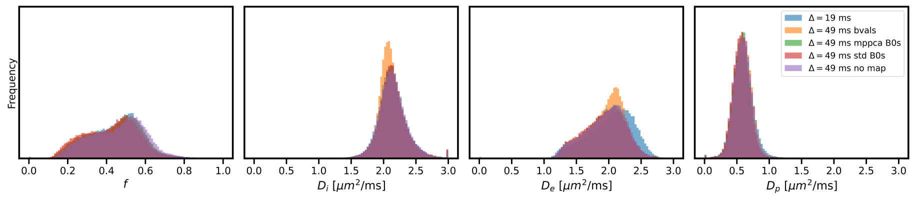

Figure S7: Histograms of the SM parameter distributions using various settings and datasets.

7 Simulations with in vivo protocol

To evaluate SM fitting with different (non-optimized) protocols, simulations were performed in the same manner as described in Section 2.5 of the main manuscript, but using the in vivo protocol with multiple B-tensors for forward signal simulation. The results in Fig. S9 show a slight decrease in performance compared to Fig. S3. The INR method, however, still demonstrates superior performance relative to the other methods.

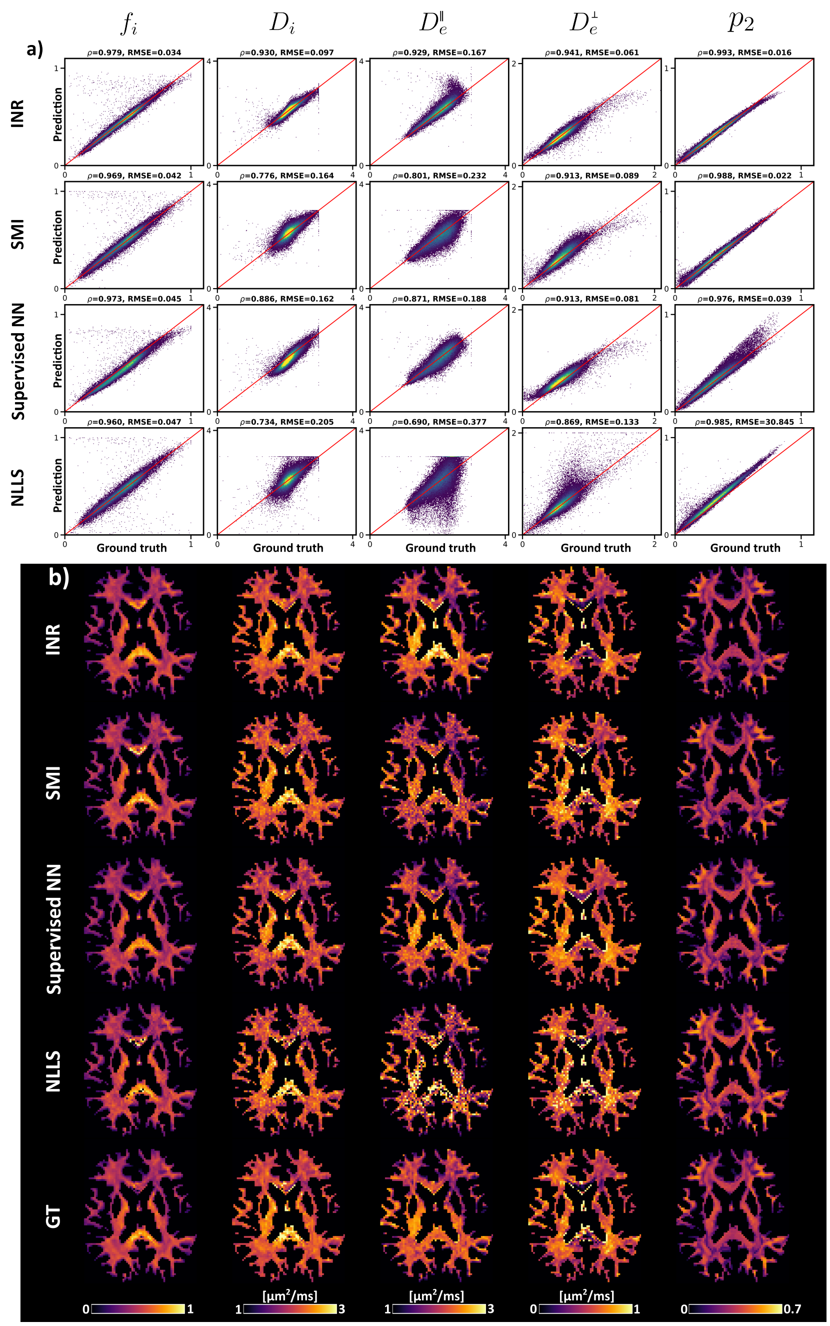

Figure S8: Experiment 1 (SNR 50) without applying smoothing to the ground truth parameters: **a)** Scatter density plots of ground truth versus parameter estimations of all methods. The titles of the subplots indicate ρ and RMSE. **b)** SM parameter maps corresponding to the results in **a**. Bottom row shows the ground truth (GT).

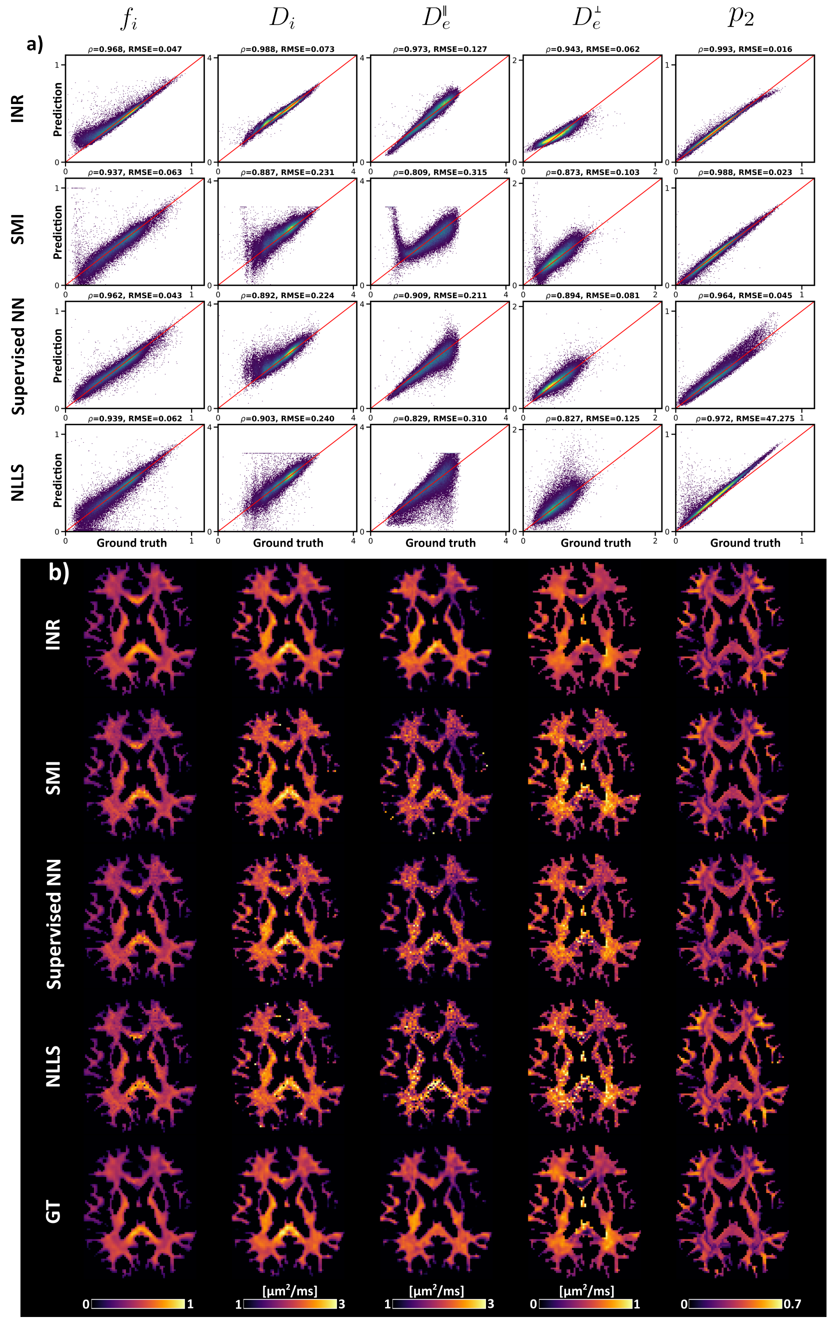

Figure S9: Fitting methods comparison with in vivo protocol (SNR = 50). Scatter density plots of the ground truth versus the parameter estimations of all methods. Row numbers correspond to method numbering from section 2.8.1 of the main document. Title of the subplots indicate ρ and RMSE. **b)** SM parameter maps corresponding to the results in **a**. Bottom row shows the ground truth (GT).

References

[1] Tancik, M. *et al.* Fourier features let networks learn high frequency functions in low dimensional domains. *Advances in neural information processing systems* **33**, 7537–7547 (2020).
