# Supplementary material for: Implicit neural representations for accurate estimation of the Standard Model of white matter
Source: Commun Biol. 2025 Dec 24;9:120. doi: 10.1038/s42003-025-09399-5 (PMC12852660; doi:10.1038/s42003-025-09399-5)
Supplement: Supplementary file 2 — Reporting Summary [file 42003_2025_9399_MOESM2_ESM.pdf]

nature portfolio

Corresponding author(s): Tom Hendriks

Last updated by author(s): 12/4/2025

Reporting Summary

Nature Portfolio wishes to improve the reproducibility of the work that we publish. This form provides structure for consistency and transparency in reporting. For further information on Nature Portfolio policies, see our Editorial Policies and the Editorial Policy Checklist.

Statistics

For all statistical analyses, confirm that the following items are present in the figure legend, table legend, main text, or Methods section.

n/a	Confirmed	
☒	☐	The exact sample size (*n*) for each experimental group/condition, given as a discrete number and unit of measurement
☒	☐	A statement on whether measurements were taken from distinct samples or whether the same sample was measured repeatedly
☒	☐	The statistical test(s) used AND whether they are one- or two-sided<br>*Only common tests should be described solely by name; describe more complex techniques in the Methods section.*
☒	☐	A description of all covariates tested
☒	☐	A description of any assumptions or corrections, such as tests of normality and adjustment for multiple comparisons
☒	☐	A full description of the statistical parameters including central tendency (e.g. means) or other basic estimates (e.g. regression coefficient) AND variation (e.g. standard deviation) or associated estimates of uncertainty (e.g. confidence intervals)
☒	☐	For null hypothesis testing, the test statistic (e.g. *F*, *t*, *r*) with confidence intervals, effect sizes, degrees of freedom and *P* value noted<br>*Give P values as exact values whenever suitable.*
☒	☐	For Bayesian analysis, information on the choice of priors and Markov chain Monte Carlo settings
☒	☐	For hierarchical and complex designs, identification of the appropriate level for tests and full reporting of outcomes
☒	☐	Estimates of effect sizes (e.g. Cohen's *d*, Pearson's *r*), indicating how they were calculated

Our web collection on statistics for biologists contains articles on many of the points above.

Software and code

Policy information about availability of computer code

Data collection	Python 3.10, Matlab R2021b
Data analysis	Python 3.10, Pytorch 2.0, SciPy 1.16.1, sklearn 1.7.1 Matplotlib, detailed description of all packages used, and all custom made code are available on our GitHub

For manuscripts utilizing custom algorithms or software that are central to the research but not yet described in published literature, software must be made available to editors and reviewers. We strongly encourage code deposition in a community repository (e.g. GitHub). See the Nature Portfolio guidelines for submitting code & software for further information.

Data

Policy information about availability of data

All manuscripts must include a data availability statement. This statement should provide the following information, where applicable:

- Accession codes, unique identifiers, or web links for publicly available datasets
- A description of any restrictions on data availability
- For clinical datasets or third party data, please ensure that the statement adheres to our policy

The MGH Connectome Diffusion Microstructure Dataset is publicly available. The synthetic dataset is available. The in-vivo dataset can be made available upon reasonable request

Research involving human participants, their data, or biological material

Policy information about studies with human participants or human data. See also policy information about sex, gender (identity/presentation), and sexual orientation and race, ethnicity and racism.

Reporting on sex and gender	There is no reporting on sex and gender, as this is of no importance for this research
Reporting on race, ethnicity, or other socially relevant groupings	There is no reporting on race ethnicity, or other groupings, as this is of no importance for this research
Population characteristics	There is no study population
Recruitment	The MGH Connectome Diffusion Microstructure Dataset is a publically available dataset, the in-vivo data is a single healthy volunteer recruited within the lab
Ethics oversight	N/A

Note that full information on the approval of the study protocol must also be provided in the manuscript.

Field-specific reporting

Please select the one below that is the best fit for your research. If you are not sure, read the appropriate sections before making your selection.

☒ Life sciences ☐ Behavioural & social sciences ☐ Ecological, evolutionary & environmental sciences

For a reference copy of the document with all sections, see nature.com/documents/nr-reporting-summary-flat.pdf

Life sciences study design

All studies must disclose on these points even when the disclosure is negative.

Sample size	There are no group comparisons in this study
Data exclusions	There is no excluded data in this study
Replication	N/A
Randomization	N/A
Blinding	N/A

Reporting for specific materials, systems and methods

We require information from authors about some types of materials, experimental systems and methods used in many studies. Here, indicate whether each material, system or method listed is relevant to your study. If you are not sure if a list item applies to your research, read the appropriate section before selecting a response.

Materials & experimental systems

n/a	Involved in the study	
☒	☐	Antibodies
☒	☐	Eukaryotic cell lines
☒	☐	Palaeontology and archaeology
☒	☐	Animals and other organisms
☒	☐	Clinical data
☒	☐	Dual use research of concern
☒	☐	Plants

Methods

n/a	Involved in the study	
☒	☐	ChIP-seq
☒	☐	Flow cytometry
☐	☒	MRI-based neuroimaging

Plants

Seed stocks

Report on the source of all seed stocks or other plant material used. If applicable, state the seed stock centre and catalogue number. If plant specimens were collected from the field, describe the collection location, date and sampling procedures.

Novel plant genotypes

Describe the methods by which all novel plant genotypes were produced. This includes those generated by transgenic approaches, gene editing, chemical/radiation-based mutagenesis and hybridization. For transgenic lines, describe the transformation method, the number of independent lines analyzed and the generation upon which experiments were performed. For gene-edited lines, describe the editor used, the endogenous sequence targeted for editing, the targeting guide RNA sequence (if applicable) and how the editor was applied.

Authentication

Describe any authentication procedures for each seed stock used or novel genotype generated. Describe any experiments used to assess the effect of a mutation and, where applicable, how potential secondary effects (e.g. second site T-DNA insertions, mosiacism, off-target gene editing) were examined.

Magnetic resonance imaging

Experimental design

Design type: Resting state

Design specifications: One block

Behavioral performance measures: N/A

Acquisition

Imaging type(s): Diffusion MRI

Field strength: 3T

Sequence & imaging parameters: The MGH Connectome Diffusion Microstructure Dataset is described extensively in Tian (Scientific Data, 2022). The in-vivo dataset: Spin-echo, EPI, TE 77, TR 2800, voxel size 3x3x3, FOV 210x210x45

Area of acquisition: Whole brain for MGH, 15x3mm slices for in-vivo of the cerebrum

Diffusion MRI: ☒ Used ☐ Not used

Parameters: The MGH Connectome Diffusion Microstructure Dataset is described extensively in Tian (Scientific Data, 2022). The in-vivo dataset has 354 acquisitions, spread out over 12 different b-values, with 4 different b-tensor shapes. Details found in Table 2.

Preprocessing

Preprocessing software: The MGH Connectome Diffusion Microstructure Dataset is described extensively in Tian (Scientific Data, 2022). The in-vivo dataset was preprocessed using MRtrix3 3.0.6

Normalization: Before fitting the model the data is normalized by dividing by the 95th percentile value of the b0 images

Normalization template: Subject space

Noise and artifact removal: The in vivo data was corrected for Gibbs ringing, signal drift, motion and eddy current correction, susceptibility correction, and gradient non-uniformity image distortion and B-matrix correction

Volume censoring: A brain mask was calculated using Freesurfer

Statistical modeling & inference

Model type and settings: An implicit neural representation fit on a single acquisition

Effect(s) tested: In the synthetic datasets: reconstruction accuracy of the ground-truth data

Specify type of analysis: ☐ Whole brain ☒ ROI-based ☐ Both

Anatomical location(s): A white matter mask is applied, which is obtained from the T1 weighted image.

Statistic type for inference (See Eklund et al. 2016): Voxel wise, Pearson's correlation

Correction	None

Models & analysis

n/a	Involved in the study
☒	☐ Functional and/or effective connectivity
☒	☐ Graph analysis
☒	☐ Multivariate modeling or predictive analysis
